# Effects of Mental Impression

**Published:** 1890-03

**Authors:** 


EFFECTS OF MENTAL IMPRESSION.—A curious case of traumatic hysteria was recently reported by Dr. William C. Thompson. The patient, a man fifty years old, had seen an Italian killed by a shock from an electric wire. Two weeks later he was struck on the head by a cut wire; he at once grasped it in his hand and fell down, only recovering his senses several hours later, after having been taken to the hospital. He was then found to have right hemiplegia and hemianesthesia, and all the symptoms of typical hysteria. The wire that he had struck was a "dead" one; that is, no current was passing through it at the time of the accident; the blow had also been but slight, causing no concussion.—*Boston Journal of Health.*

